# Utilization of Carbon-Based Nanomaterials and Plate-Fin Networks in a Cold PCM Container with Application in Air Conditioning of Buildings

**DOI:** 10.3390/nano12111927

**Published:** 2022-06-04

**Authors:** Farhad Afsharpanah, Goshtasp Cheraghian, Farzam Akbarzadeh Hamedani, Elham Shokri, Seyed Soheil Mousavi Ajarostaghi

**Affiliations:** 1 Mechanical Engineering Department, Babol Noshirvani University of Technology, Babol 47148, Iran; soheilmousavi67@gmail.com; 2 Technische Universität Braunschweig, 38106 Braunschweig, Germany; 3 Mechanical Engineering Department, Chabahar Maritime and Marine University, Chabahar 99717, Iran; farzam.akbarzadeh@gmail.com; 4 Department of Architecture and Energy, Ilam University, Ilam 69315, Iran; shokry_elham@yahoo.com

**Keywords:** refrigeration, nanoparticles, ice storage system, PCM, thermal energy storage, ice-on-coil, computational fluid dynamics, CFD, heat transfer enhancement, heat exchanger

## Abstract

Cold energy storage devices are widely used for coping with the mismatch between thermal energy production and demand. These devices can store cold thermal energy and return it when required. Besides the countless advantages of these devices, their freezing rate is sluggish, therefore researchers are continuously searching for techniques to improve their operating speed. This paper tries to address this problem by simultaneously combining a network of plate fins and various types of carbon-based nanomaterials (NMs) in a series of complex computational fluid dynamics (CFD) simulations that are validated by published experimental results. Horizontal, vertical, and the combination of these two plate-fin arrangements are tested and compared to the base model. Subsequently, several carbon-based NMs, including SWCNT, MWCNT, and graphene-oxide NMs are utilized to further improve the process. The influence of these fin networks, nanoparticle types, and their volume- and mass-based concentrations within the PCM container are studied and discussed. According to the results, carbon-based NMs exhibit superior performance compared to metal-oxide NMs, so that at identical NM volume and mass fractions, MWCNT particles present a 2.77% and 17.72% faster freezing rate than the CuO particles. The combination of plate-fin network and MWCNT particles is a promising technique that can expedite the ice formation rate by up to 70.14%.

## 1. Introduction

The energy crisis is the result of a considerable bottleneck in the supply of the demanded energy resources. The world has experienced energy crises over short times in previous years, and the abysmal influence of this even short durations of energy shortage is well-known by academicians [1]. Energy storage is introduced as the solution to this problem. When energy storage devices are utilized, a bottleneck does not occur on the supply side because either the energy demand is managed and aligned by the energy supply capacity over the entire day (for example by installing domestic energy storage units in houses) or the energy capacity is stored by the supply side (for instance, by pumping the water to an elevated area during low-peak hours) to supply a considerable amount of energy when needed [2]. Since buildings account for a considerable amount of overall energy consumption (more than 10.3%) [2], the use of energy storage devices in buildings [3] can be considered a major step in balancing energy consumption and demand. Even in some cases, these devices may allow obtaining a zero-energy building [4], which operates based on renewable energies that might not be always available. Storage of energy can be carried out in different forms, such as chemical, hydraulic, and thermal. The latter can be also categorized into two groups of sensible and latent. Aside from the greater level of energy density, the latent form does not require a considerable amount of temperature difference to store energy, and since the thermal loss of a unit is directly affected by the temperature difference with the environment, these systems tend to have better efficiencies compared to the devices which use sensible form. The latent form of storing energy can be performed with the intention of cooling or heating. There are various types of cold latent energy storage systems (CES). Ice storage is one type of CES, which has lots of applications in the industry. In these units, water is employed as the PCM, and an external tubular fluid flow, namely, the heat transfer flow (HTF) is used to charge and discharge the system. Similar to the other PCMs, water suffers from low values of thermal conductivity, and different approaches have been taken by the researchers to address this issue, many of which are widely used for conventional heat transfer enhancement problems. Employing porous materials [5], fins [6], turbulators [7], particles with high thermal conductivities [8], and special tube types [9] are some of the most well-known techniques for this purpose.

In the experimental/numerical study of Labihi et al. [10], the influence of incorporating PCM into the building walls for energy preservation was investigated. They integrated encapsulated PCM in a test setup that included a vertical wall. The implementation of PCM in the wall could stabilize the indoor temperature, also it was able to delay heat transfer from the wall and improve the indoor temperature in the range of 24–26 °C. They studied the impacts of various parameters, such as the type, position, and thickness of the PCM layer. They introduced PCM as a smart insulation layer, since it has a low thermal conductivity, and can passively manage the indoor temperature. According to their results, placing the PCM closer to the indoor environment leads to better thermal results. They concluded that this method leads to up to 15% energy saving compared to the conventional insulation techniques. Mahmoud et al. [11] worked on the melting enhancement of RT-35 wax in a triple-tube (TT) unit with sloped circular fins. They evaluated the influence of fins with various sizes and orientations with the same fin volume fractions. The results indicated that installing this type of fin with a downward–upward pattern can raise the melting rate by up to 88%. Sun et al. [12] employed inline and staggered arrangements of circular fins to improve the rate of the phase change process. They discovered that the staggered distribution of fins can improve the melting and heat charging rate by 37.2 and 59.1%, respectively. Amer et al. [13] examined the melting process in a PCM-filled shell-and-tube (SAT) unit with a various number of circular fins. They showed that with 36, 30, 18, and 6 fins, 72, 72, 66, and 53% of melting rate enhancement can be obtained compared to the base SAT unit without fins. They presented the optimal size and number of fins for their geometry. They maintained the volume of fins throughout their study.

In the work of Iasiello et al. [14], the melting process of foam-embedded paraffin wax was examined using both experimental and computational techniques. They used the finite element method to model the process in various hypergravity states. They revealed that at higher gravity accelerations, the natural convection is considerably intensified. Bianco et al. [15] used the Pareto optimization technique to optimize the performance and cost-effectiveness of a finned heat sink integrated with PCM. Based on their results, a lower device length and a thicker fin design can reduce the operation time. They also obtained the optimal design parameters for this system. In the research conducted by Ghahremannezhad et al. [16], the utilization of non-homogeneous porous foams as heat transfer enhancers in energy storage systems was comprehensively investigated. They compared porous materials with gradient pore structures against conventional ones which have a uniform pore structure. Both negative and positive structure gradients were examined and their effect was evaluated on the thermal response of an energy storage system. They showed that these gradient porous structures can present a more uniform melting profile. Huang et al. [17] performed research on PCM-integrated finned metal foam heat sinks. They investigated the influence of pore density and porosity. They recommended heat sinks with larger pore densities and lower porosities for practical applications. The simultaneous use of porous foams and fins was also the subject of research conducted by Guo et al. [18], and Yang et al. [19].

Fertelli et al. [20] performed numerical simulations to study the impact of HTF cylinder position in a cavity on the freezing process. Two different models including one and four cylinders were considered. For the one-cylinder model, the vertical placement of the cylinder showed no significant impact on the freezing process. However, for the four-cylinder case, the highest freezing performance was achieved when the distance between the cylinder and the top and bottom surfaces of the storage tank was equal to 2d, where d is the diameter of the cylinder. Wang et al. [21] studied the discharge process of an external melt ice storage system. They evaluated the influence of various parameters such as the initial amount of ice, the place of the hot fluid inlet, and the flow rate and temperature of the HTF. According to their results, the entrance of the hot water flow from the bottom and its exit from the top of the storage can preserve the thermal stratification and present a lower temperature at the outlet.

Abdelrahman et al. [22] experimentally analyzed the freezing and melting operations in a CES unit that consisted of two concentric coils with helical profiles. They selected a single geometry for their research and solely concentrated on the HTF flow rate and temperature. Their study revealed that while the impact of HTF temperature is critical to the process, the effects of the flow rate are not notable. Zohra et al. [23] integrated two various PCM types with different melting temperatures to configure the phase change temperature near the desired point. They designed the unit in a cubic shape in which the two PCM were separated by a diagonal aluminum plate. They also considered the influence of heat radiation in their study. They showed that after the phase change of the first PCM, the temperature increases until it reaches the melting temperature of the second PCM. At that point, the temperature rise stops, and heat is absorbed by the second PCM to complete the melting process. Their discussions showed that with this system, the overall energy production and storage rates are elevated by up to 20 min. Tiari et al. [24] conducted a comprehensive computational study to examine the influence of various annular fins on the acceleration of both charging and discharging processes in an SAT TES. They maintained the fin volume for all of their studied cases. They used an innovative pattern for the distribution of fins where the fin length was not uniform. Their assessments showed that with 20 non-uniform fins with higher lengths in the bottom of the unit and lower lengths in the top of the unit the charging rate is improved by up to 74%. However, they showed that the uniform type of fin is more efficient during the discharging process by exhibiting virtually 79% of enhancement. By considering both these processes, they recommended the uniform-length fins.

As was stated earlier, the dispersion of particles with high thermal conductivity can elevate the low thermal conductivity of PCMs. While the addition of milli- or micrometer-sized particles can lead to sedimentation of the particles, due to their relatively small surface area and large size, smaller-sized nanomaterials (NMs) additives have properly settled this issue [25]. Even though nanomaterials have widespread application and many advantages in the industry [26,27], some research studies have discussed alarming health problems that might be caused by some of these NMs. For instance, the study of Karlsson et al. [28] compares the cytotoxicity and DNA damage by some NMs, including those that are widely used in thermal engineering applications, such as TiO_2_ and CuO. According to their finding, the toxicity level of the NMs is highly dependent on the NM type. Among the tested NMs, the cytotoxicity and DNA damage caused by CuO NMs were more severe. On the other hand, carbon-based NMs present a far lower toxicity level compared to this widely used metal-oxide NM. Moreover, in thermal and energy storage applications their performance is promising [29].

The melting process of a mixture of n-Octadecane and Al_2_O_3_ NMs was studied in the work of Bouzennada [30]. They modeled the process using the Comsol-Multiphysics software. They evaluated the effects of the HTF tube position and nanoparticle concentration. Their observations revealed that moving the HTF tube toward the bottom of the unit improves the melting rate. Afsharpanah et al. [31] investigated the geometric optimization in an SAT CES by the means of the computational fluid dynamics (CFD) technique. Four of the parameters were focused on the heat exchanger (HX), while two parameters were concentrated on improving the fin. They also enhanced the flow parameters. They extended this research by adding metal-oxide NMs and revealed that the simultaneous use of cylindrical plate fins and CuO NMs with a fraction of 3% reduce the full charging rate by around 30% [32]. Afsharpanah et al. [33] presented a numerical investigation of the geometrical and flow parameter improvement in a cuboid shape ice storage unit with serpentine HTF tubes. They assessed various dimensionless variables on the stored energy ratio. The basis of this research is the optimized flow and geometric characteristics of their study, the specifications of which will be discussed further in this paper.

As discussed in the literature review, improving the sluggish phase change rate in the CES devices is a necessity for the generalization of these units. To address this, at first, this study investigates the enhancement obtained by installing several networks of horizontal and vertical plate fins on the serpentine HTF tubes of a cuboid-shaped CES unit, then, to further improve the process, several nanomaterial-based solutions are tested as alternatives to the pure PCM. Since the CES devices are usually employed in areas such as refrigeration units or air conditioning systems, instead of metal-oxide particles such as CuO which have been proved to have a higher chance of creating cytotoxicity and DNA damage, some carbon-based alternatives are employed. These NMs are single-walled (SWCNT) and multi-walled (MWCNT) carbon nanotubes and graphene-oxide particles. The study involves a series of complex computational simulations to improve the ice formation in the container. During the study, four patterns of fin network, including the finless case, and cases with horizontal fins, vertical fins, and a combination of horizontal and vertical fins are examined. Moreover, the influence of each of the above-mentioned NMs is studied and compared to their metal-oxide rival, CuO. After choosing the optimal particles regarding the solidification rate, several concentrations of these NMs are tested. The findings are presented in the form of plots and solid fraction contours and will be thoroughly discussed in the following sections.

## 2. Computational Model

This part of the paper is devoted to explaining the procedure used for obtaining the CFD model.

### 2.1. Demonstration and Physical Representation

**Figure 1:**
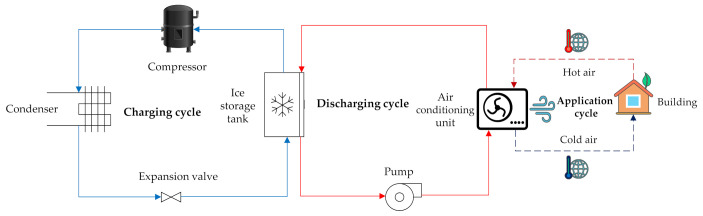
The thermodynamic cycles of charging, discharging, and application of the CES unit.

Considering the importance of balancing the energy demand, this work concentrates on the improvement of the charging process in a cuboid-shaped CES unit which has applications in the air conditioning of buildings. To better understand the process, Figure 1 demonstrates three thermodynamic cycles which are related to charging, discharging, and application of this CES unit.

As can be observed in the figure, a conventional compression refrigeration cycle is employed to charge several CES units when the power network is not under a significant amount of load (for instance, the interval between midnight and morning). The unit is then discharged during the peak hours, and since the unit is coupled with the air conditioning unit, it can provide cold thermal energy with a low amount of electricity (a water pump and a fan), meaning that during the peak hours, the air conditioning systems can operate without the need of compression refrigeration. This considerably reduces the power consumption during peak hours.

**Figure 2:**
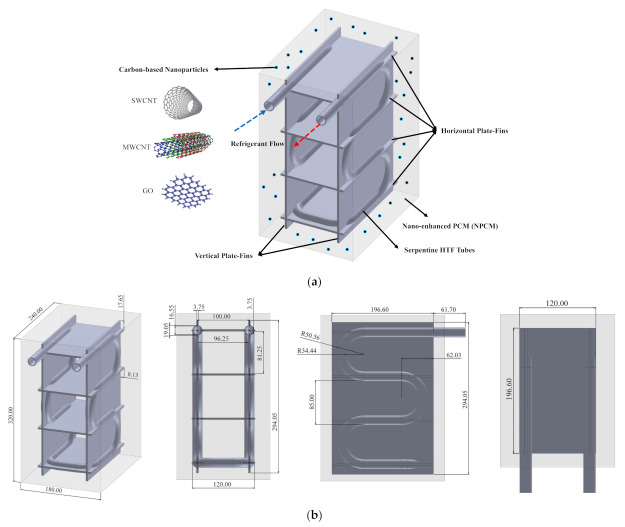
The CES container: (**a**) the physical representation; (**b**) complete geometric sizes.

Figure 2 visually illustrates the geometrical representation as well as the size of various parts of the CES unit which is a cuboid-shaped ice storage bank. The current study aims to report the impacts of horizontal and vertical plate-fin networks and also carbon-based NMs on enhancing the phase change process in this unit. As can be observed, the unit includes five components:

1. The shell-side of the cuboid container is full of a PCM, which is desalinated water (H_2_O). The PCM solidifies as the refrigerant coils draw heat from the unit;
2. The horizontal plate-fin network includes four horizontal fins that horizontally connect the serpentine tubes to increase their influence region;
3. The vertical plate-fin network includes two vertical fins that vertically connect the serpentine tubes to use thermal conduction for acquiring a better thermal distribution;
4. The refrigerant serpentine tubes are employed as the passage for HTF flow, which is an ethylene alcohol/water (C_2_H_6_O_2_/H_2_O 30%vol) solution with a Reynolds number and temperature of 1500 and −10 °C;
5. Carbon-based NMs are homogeneously distributed within the PCM to improve its thermal behavior.

As was discussed before, the base geometry of the tubes and container is according to the optimal unit of Afsharpanah et al. [33], and here plate-fin networks and NMs are added to provide an even better solidification rate. As recommended in previous studies [32], copper C11000 alloy is chosen as the material of the fin networks and tubes. Regarding the NMs, GO, SWCNT, and MWCNT carbon-based NMs are tested and compared to one of the most widely used metal-oxide NMs, CuO. GO NMs are in the form of a brown powder, with layered-shaped particles, dimensions in the range of 0.6–1.2 micrometer, and a thickness between 0.6 and 1.2 nm. They have oxygen and carbon percentages of 31.95 and 68.04%, respectively. Both SWCNT and MWCNT NMs are solely made of carbon atoms and they possess black powder forms with the shape of hollow tubes made of graphite layers. Their difference is in the number of these concentric layers. If it is a single layer of graphite layer, it is known as SWCNT while MWCNTs are made of several concentric layers. The axial length of SWCNTs and MWCNTs range in 15–50 and 10–30 nm while their diameter is 1.2 and 20–30 nm, respectively. The shape of these NMs is graphically illustrated in Figure 2a.

The thermophysical data that are needed for running the simulations are presented in Table 1.

To address the impacts of the parameters which is the goal of this study, altogether, fourteen cases are specified that are tabulated in Table 2. The research variables include the plate-fin network arrangements, NM type at identical NM volume and mass fractions, and NM volume fraction. The base geometry of these cases are identical; however, vertical and horizontal plate-fin networks are excluded in some of the cases.

The computational simulations make use of the following list of assumptions:

1. The PCM possesses different properties before and after the phase change;
2. The fluid flow in the tubes and shell are laminar and incompressible;
3. The simulations are unsteady;
4. The walls of the cuboid-shaped shell are well-insulated;
5. The buoyancy effects are contemplated through the Boussinesq approximation;
6. By the means of ultrasonication and homogenization techniques, the NMs are well-distributed on the shell side, and the mixture of PCM and NMs is homogenous.

### 2.2. Methodology, Equations, Boundary, and Initial Conditions

The current research employs the CFD method (with finite-volume codes) for modeling the phase change progress. To perform the analysis, after creating the geometries in the ANSYS SpaceClaim 22R1 software (Canonsburg, PA, USA) they were transferred to ANSYS meshing 22R1 software (Canonsburg, PA, USA), where the continuous geometry domains were broken down into small cells for which the computational processes were carried out in the ANSYS Fluent 22R1 software (Canonsburg, PA, USA) based on the governing correlations. To include the influence of the nanomaterials, by the means of a user-defined function (UDF) within the ANSYS Fluent software, and also by employing the homogenous model [34], the properties of the nano-enhanced PCM (NPCM) were substituted instead of the pure PCM. Based on these properties, the simulations were carried out for each time step until the termination criteria were met, then the computations for the next time-step were started. These criteria were set to be the residuals of 10^−4^ for continuity and velocities and 10^−6^ for the energy correlation. The governing correlations continued to be solved for successive time steps until the solid fraction reached the value of 99%.

The governing correlations which were solved throughout the simulations are presented as follows:

Since in most cases of the current research, NMs are distributed within the PCM, it is necessary to acquire the properties of these NPCMs according to the data of pure PCM (PCM), and nanomaterials (NMs). Therefore, the following correlations are employed [32]:

(1) $$\rho_{NPCM}=(1-\psi )\rho_{PCM}+\psi \rho_{nm}$$

(2) $${\left(\rho c_{p}\right)}_{NPCM}=(1-\psi ){\left(\rho c_{p}\right)}_{b}+\psi {\left(\rho c_{p}\right)}_{np}$$

(3) $$\frac{k_{NPCM}}{k_{PCM}}=\frac{\left(k_{nm}+2k_{PCM}\right)-2\psi \left(k_{PCM}-k_{nm}\right)}{\left(k_{nm}+2k_{PCM}\right)+\psi \left(k_{PCM}-k_{nm}\right)}$$

(4) $${\left(\rho h_{sf}\right)}_{NPCM}=(1-\psi ){\left(\rho h_{sf}\right)}_{b}$$

(5) $$\mu_{NPCM}=\frac{\mu_{PCM}}{{\left(1-\psi \right)}^{2.5}}$$

where ψ is the NM volume fraction and correlates with the NM mass fraction (ω) by the following equation:

(6) $$\omega =\frac{\rho_{nm}.\psi }{\rho_{PCM}.(1-\psi )+\rho_{nm}.\psi }$$

The continuity, momentum, and energy correlations write [35]:

(7) $$\nabla \cdot \vec{v}=0$$

(8) $$\frac{\partial v}{\partial t}+\vec{v}\cdot \nabla \vec{v}=\frac{1}{\rho }\left(-\nabla \rho +\mu \nabla^{2}\vec{v}+\rho \beta \left(T-T_{reference}\right)\right)-\frac{{\left(1-\lambda \right)}^{2}}{\lambda^{3}}C_{mushy}\vec{v}$$

(9) $$\frac{\partial h_{total}}{\partial t}+\nabla \cdot \left(\vec{v}h_{sensible}\right)=\nabla \cdot \left(\frac{k}{\rho c_{p}}\nabla h_{sensible}\right)$$

In which, $C_{mushy}$ possesses the value of 100,000 kg/s·m^3^ and indicates the mushy zone constant. In the above equation, $h_{latent}$ and $h_{\mathrm{sensible}}$, and their summation are defined as:

(10) $$h_{total}=h_{sensible}+h_{latent}\to \;\left\{\begin{array}{l} h_{\mathrm{sensible}}=h_{reference}+c\int_{T_{reference}}^{T}dT\\ h_{latent}=\sum_{i=1}^{n}h_{sf}.(1-\kappa_{i}) \end{array}\right\}$$

The enthalpy-porosity method was utilized to address the heat transfer throughout the charging progress. The κ variable in the previous correlation represents the solid fraction, which varies in the range of 0≤κ≤1. The value of κ for a cell specifies its situation among liquid (κ=0), mushy-zone (0<κ<1), and solid (κ=1) states. The cells with a mushy-zone state resemble a porous medium, the porosity of which is 1−κ. This parameter is defined as [36]:

(11) $$\kappa =\left\{\begin{array}{ll} 1-\frac{T-T_{solidus}}{T_{\mathrm{liquidus}}-T_{solidus}} & if\;T_{solidus}<T<T_{\mathrm{liquidus}}\\ 1 & if\;T\leq T_{solidus}\\ 0 & if\;T\geq T_{\mathrm{liquidus}} \end{array}\right\}$$

Finally, to include the influence of buoyancy on the shell side, the Boussinesq approximation was employed:

(12) $$\rho =\rho_{0}{\left[\beta \left(T-T_{liquidus}\right)+1\right]}^{-1}$$

In which, β with a value of −6.73 × 10^−5^ 1/K is the thermal expansion coefficient. Finally, to calculate the heat flux passing through the coolant tube walls, the following equation based on the Fourier’s law was utilized:

(13) $$\delta_{r}=-k_{tube}\frac{dT(r)}{dr}$$

These start-up and boundary conditions were imposed:

A static velocity field with a temperature of +0.5 °C was chosen as the start-up state of the unit. For the outlet and inlet of the refrigerant flow, pressure ($P_{rel}=0\;Pa$) and velocity ($v_{inlet}=0.4623,\;T_{inlet}=-10$ °C) boundaries are imposed.

### 2.3. Validation of the Numerical Method

**Figure 3:**
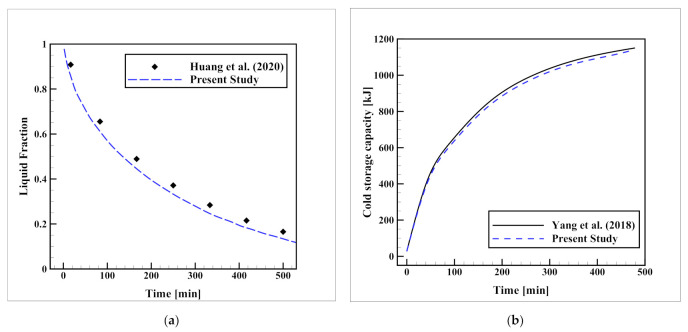
Validation of the CFD method with (**a**) the experimental results from the study of Huang et al. [37], and (**b**) the numerical data of Yang et al. [38].

To make sure that the simulations are on the right track, performing a validation analysis is necessary. For this purpose, two separate validation analyses were carried out. In the first step, due to the lack of similar experimental geometry, the experimental setup of Huang et al. [37], which was focused on the solidification progress of water in a shell-and-tube (SAT) ice bank is computationally simulated as a three-dimensional model. The reason for selecting this setup is that this setup, involves an identical PCM and HTF material (water and ethylene glycol, respectively) and similarly, it includes fins, thereby this setup can be a comprehensive basis that involves water solidification, ethylene glycol flow, and thermal conduction in metallic fins. The examined SAT ice bank had a length and diameter of 145 and 170 mm respectively. Inside, there was an aluminum finned HTF pipe with outer and inner diameters of 50 and 40 mm, respectively, carrying ethylene glycol flow at $T_{inlet}=-10$ °C. The pipe had six axial fins, the width, and length of which were 5 and 50 mm, respectively. The comparison of their empirical data with the current simulation results is plotted in Figure 3a. The close compatibility of the results with the experimental data with the maximum deviation of 5.1% verifies the validity of the CFD method. In the next stage, to use a more similar geometry, the numerical setup of Yang et al. [38] was remodeled. Their model was an ice-on-coil ice storage unit that included a single-layer serpentine HTF tube carrying ethylene glycol solution at $T_{inlet}=-10$ °C in a thin shell that contained water as the PCM. The length, width, and thickness of the container were 1 m, 0.5 m, and 0.05 m, respectively, while a copper tube with a diameter of 12 mm was used as the HTF pipe. The comparison of the changes in the cold storage capacity over time is presented in Figure 3b, which shows excellent compatibility with a maximum deviation of 1.4%, indicating the validity of the model for a more similar geometry.

**Figure 4:**
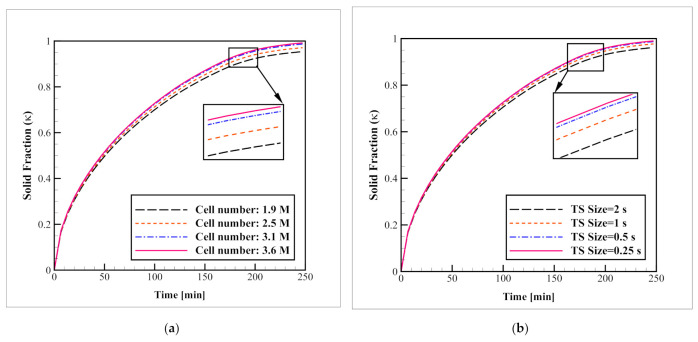
The (**a**) mesh size independence, and (**b**) time-step size tests.

**Figure 5:**
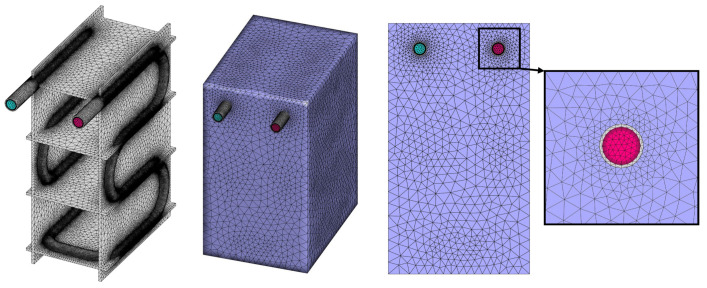
The final grid network for Case D.

To select the optimal grid size and time-step size, considering both precision and computational cost, a sensitivity analysis was carried out for the grid and time-step sizes for Case D. To do so, the simulation was repeated several times with the cell number of 1.9, 2.5, 3.1, and 3.6 millions of computational elements, and time-step sizes of 0.25, 0.5, 1 and 2 s. The progression of the average solid fraction has been illustrated in Figure 4 for each of these modes. Based on these results, a grid network with 3.1 million elements and the simulation with a time-step size of 0.5 s can complete the progress with adequate accuracy. Therefore, the simulations are continued based on these cell and time-step sizes. Figure 5 presents the mesh network that is chosen for this case. The grid network that has been illustrated in this figure was generated with cell sizes of 6, 2, and 2 mm for the NPCM, refrigerant, and tube regions, respectively. The refrigerant flow region was refined using six layers of thin boundary-layer grids with a first-layer thickness of 0.25 mm and a growth ratio of 1.15. Moreover, a proximity function was utilized to reduce the cell size where various regions are close to each other.

## 3. Results

This part of the manuscript tries to elucidate the impacts of various parameters on the progression of solid fractions during the solidification process of the unit. The examined parameters are the presence of horizontal and vertical plate-fin arrangements, the addition of different carbon-based NMs to the PCM at identical volume and mass fractions, and the volume fraction of the optimal NM. For the first parameter, cases with horizontal, vertical, and a combination of horizontal and vertical plate-fin networks are investigated and compared with the base model without plate-fin networks. The next parameter tests different carbon-based NM types including GO, SWCNT, and MWCNT, and compares them to the case with the optimal metal-oxide NM type (CuO) and the one without any NM additive, at both identical NM volume (ψ = 3%) and mass fraction (ω = 10%) conditions. After specifying the best NM for this application, the influence of various volume fractions (ψ = 0, 1, 3, and 5%) of this NM is tested.

### 3.1. Different Arrangements of Plate-Fin Networks

**Figure 6:**
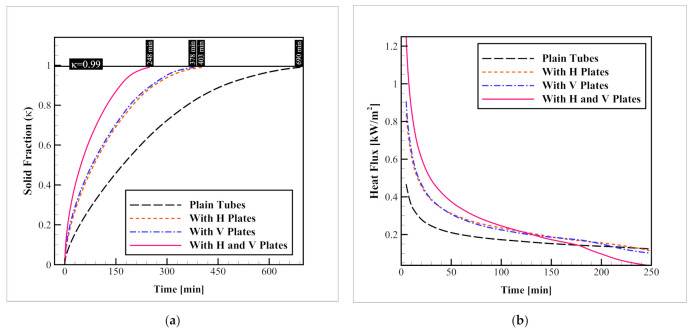
Impacts of the plate-fin network on (**a**) Progression of the average solid fraction; and (**b**) The heat flux passing through the refrigerant tubes.

This section evaluates the significance of plate-fin networks on the charging rate of the storage. Horizontal, vertical, and a combination of these two plate-fin networks are tested and compared to the finless model. The results regarding the evolution of the average solid fraction and the heat flux passing through the refrigerant tubes are obtained and illustrated in Figure 6. As indicated in the figure, while the complete solidification takes as much as 690 min, vertical and horizontal plate-fin networks reduce this time to 403 and 378 min, respectively. On the other hand, when a combination of horizontal and vertical plate-fin networks is used, the process takes only 248 min. These values indicate that compared to the base model, the presence of vertical and horizontal, and a combination of these two plate-fin networks can enhance the solid fraction progression speed up to 41.59, 45.21, and 64.05%, respectively. The enhancement of the process is due to the increased heat transfer surface within the container, which distributes the coldness of the HTF tube more properly within the container. Since the overall heat transfer area of the horizontal and vertical plate-fin networks are not much different, it can be observed that their influence on the charging rate is not significant; however, the horizontal network leads to a slightly better outcome. The same pattern is found for the heat flux alternations over time. As can be observed in the figure, the highest heat flux is obtained when the combination of vertical and horizontal plate-fin networks is utilized. The heat flux with horizontal and vertical fin arrangements are almost the same, with a slight superiority of the horizontal arrangement over the vertical one. The other point that is worth mentioning is that as time passes, the heat flux is constantly decreasing due to the formation of ice layers on the fin network and tubes which reduces the heat transfer level. Clearly, since the ice formation rate in the case with a horizontal and vertical plate-fin network is higher, this reduction is advanced toward an earlier time, which is clear in the heat flux plot at around t = 180 min. At this time the space between tubes is completely filled with ice for these cases and thus the heat flux and the heat transfer coefficient decline considerably.

**Figure 7:**
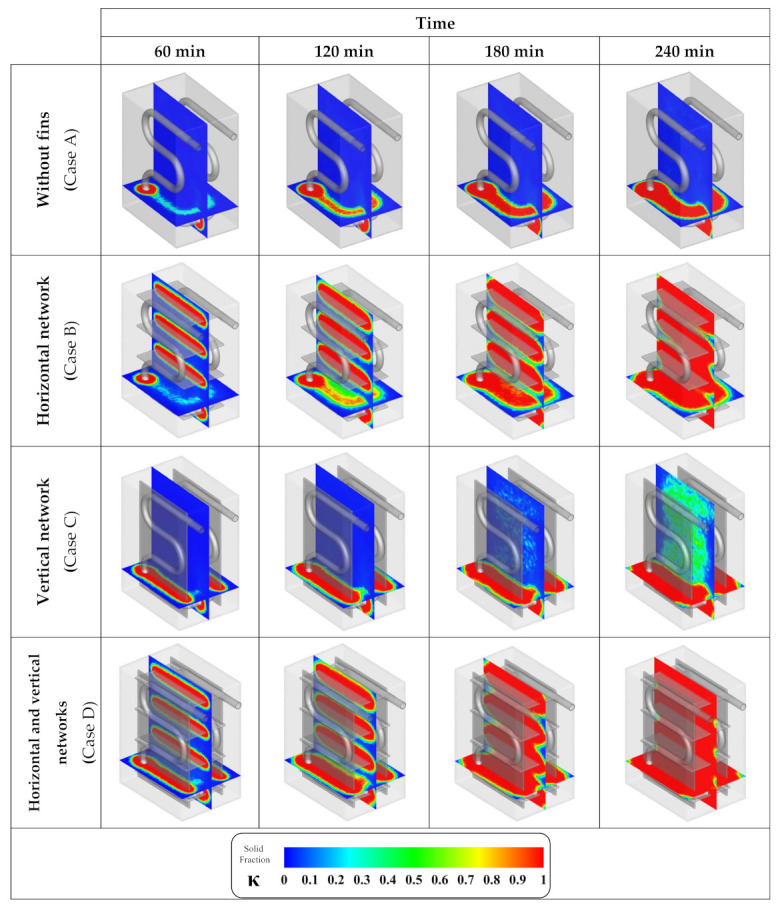
Contours of solid fraction advancement with various plate-fin arrangements.

Figure 7 presents the solid fraction contours for the cases with different plate-fin arrangements. In this figure, the blue color indicates the liquid phase while the red color belongs to the solid phase. As shown in this figure, while it was previously stated that the overall ice formation rate in the container with horizontal and vertical plate-fin networks are similar, their ice formation pattern is significantly different. This is due to different concentration areas for each of these plate-fin arrangements which consequently affects the pattern of phase change within the container. While the horizontal fins create a significant advancement in the ice formation rate in the vertical slice of the contour, the vertical fins mostly improve the ice formation in the horizontal contour slice. In general, the considerable influence of these fin networks on the solidification rate is evident.

### 3.2. Nanomaterial Type (Identical Volume Fractions)

**Figure 8:**
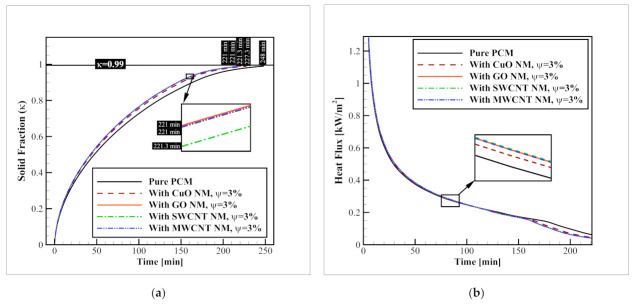
The influence of NM type at identical NM volume fractions on (**a**) progression of the average solid fraction; and (**b**) the heat flux passing through the refrigerant tubes.

To select the optimal NM for further examinations, in this section, three different NM types, including GO, SWCNT, and MWCNT, were tested and compared to the best metal-oxide NM recommended for a similar application in a previous study [32], and the benchmark model without any NM additive at identical NM volume fractions (ψ = 3%). It should be noted that in all of these cases, both horizontal and vertical fins networks are used. The results of the average solid fraction and heat flux values with each of these NM additives are illustrated in Figure 8. As indicated in this figure, while the complete solidification of the benchmark model with pure PCM takes 248 min, the addition of CuO, SWCNT, MWCNT, and GO NMs with a volume fraction of ψ = 3%, reduces this time to 227.3, 221.3, 221, and 221 min, respectively. This means that the addition of these NMs can accelerate the process by 8.34, 10.76, 10.88, and 10.88%, respectively. Aside from the clear superiority of carbon-based NMs, compared to the CuO (up to 2.77%), which is considered the optimal metal-oxide NM for this application according to a previous study [32], the results with different carbon-based NMs are similar and inconclusive, because, besides the thermal conductivity of the NMs, their other properties affect the process as well. For instance, consider the density of the NMs. Since the density of GO NMs is almost 31% higher than SWCNT particles, when an identical NM volume fraction is considered for both of these NMs, in terms of mass, a 31% higher amount of NM can be added to the container if GO NMs are selected. Therefore, to select the optimal carbon-based NM, the NM types should be examined at identical NM mass fractions as well. The changes in the heat flux obeys the same pattern as well. The heat flux values in presence of NMs increase, and this rise for carbon-based NMs is higher than that of CuO NM. Again, the results for different carbon-based NMs is almost identical and thereby, inconclusive.

### 3.3. Nanomaterial Type (Identical Mass Fractions)

**Figure 9:**
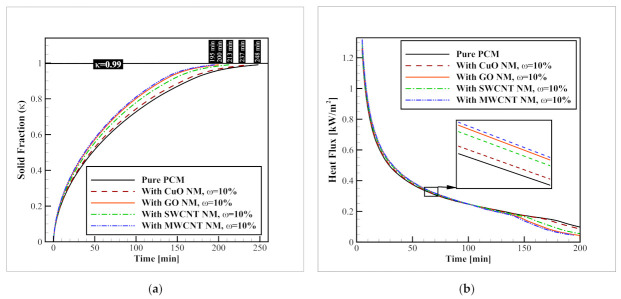
The influence of NM type at identical NM mass fractions on (**a**) progression of the average solid fraction; and (**b**) the heat flux passing through the refrigerant tubes.

In the last section, it was observed that the enhancement results for choosing the optimal carbon-based NMs are inconclusive when the NM volume fraction is held constant because the results with various carbon-based NMs are almost the same. Thereby, in this part of the manuscript, the NM mass fraction is held constant instead of the volume fraction. It means a certain mass of various NM types is added to the PCM. Once again, the results are studied on the acceleration of the average solid fraction as well as the heat flux passing through the tube walls. Figure 9 presents these results. As shown in this figure, when identical mass fractions (ω = 10%) of CuO, SWCNT, GO, and MWCNT NMs are added into the PCM, the required time for a complete solidification drops from 248 min to 237, 213, 200, and 195 min, respectively. In other words, enhancements of 4.43%, 14.11, 19.35, and 21.37%, respectively, as compared to when pure PCM is employed. Based on these values, it can be observed that the carbon-based NMs are 10.12–17.72% superior to the CuO NMs, which are considered the best metal-oxide NMs for this application based on previous work [32]. Moreover, as indicated in the introduction section, according to the study of Karlsson et al. [28] the cytotoxicity and DNA damage by CuO NMs is much more severe than those of carbon-based NMs, and this is another considerable factor that recommends the use of carbon-based NMs instead of metal-oxide NMs. Among carbon-based NMs, the best thermal performance belongs to the MWCNT NMs, and after that, GO, and SWCNT NMs have the second and third ranks in accelerating the progress (with 2.50, and 8.45% slower performance, respectively). This trend is repeated for the heat flux plots, meaning that the highest heat flux is presented when MWCNT NMs are distributed within the PCM.

Comparing these results with those that were obtained at identical volume fractions shows that while adding a certain volume of carbon-based NMs to the PCM creates a freezing enhancement that is almost independent of the NM type, the addition of a specific mass of carbon-based NM is indeed dependent on the NM type. Again, it should be noted that the geometric aspects are constant for all of these cases, and both horizontal and vertical plate-fin networks are installed on the serpentine tubes.

**Figure 10:**
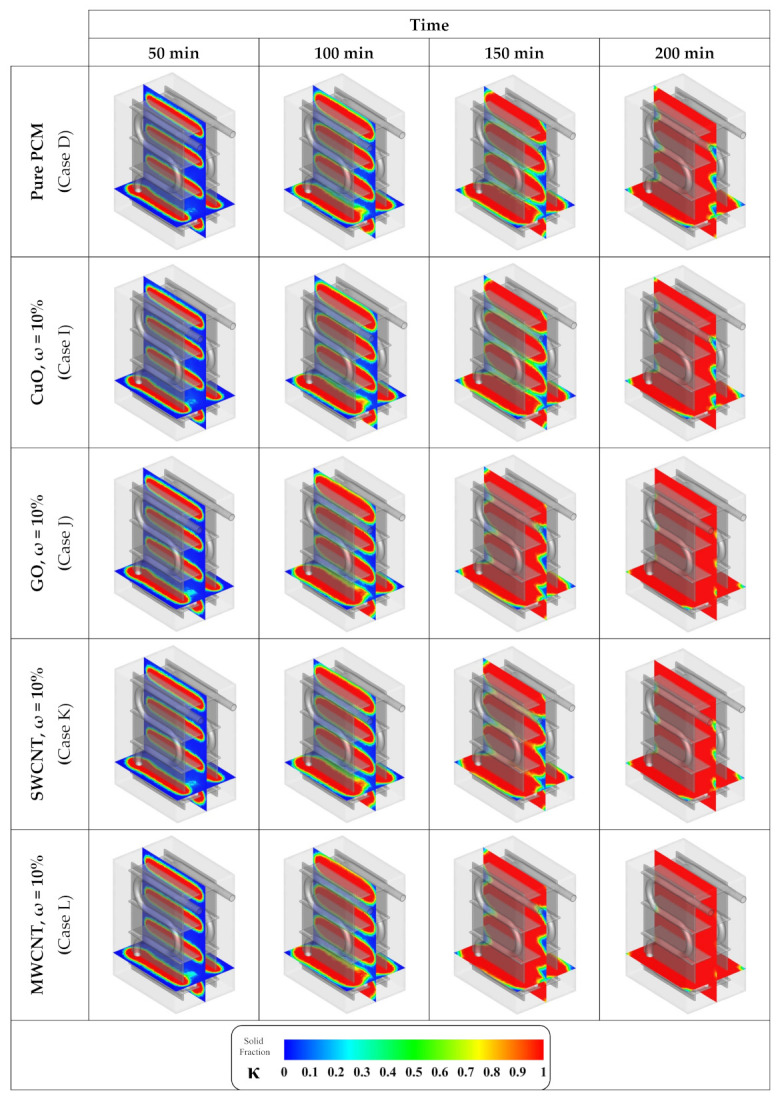
Contours of solid fraction advancement with various NM types at identical mass fractions (ω = 10%).

The solid fraction contours for different NM types at identical NM mass fractions (ω = 10%) of CuO, SWCNT, GO, and MWCNT are illustrated in Figure 10. By taking a close look at these contours, it is clear that the MWCNT NMs present the best process enhancement. While at t = 200 min, the storage is almost fully frozen in the presence of MWCNT NMs, the cases with GO, and SWCNT NMs are slightly less frozen. For the cases with CuO NMs and without NM additives, the difference is more intense.

### 3.4. Nanomaterial Volume Fraction

**Figure 11:**
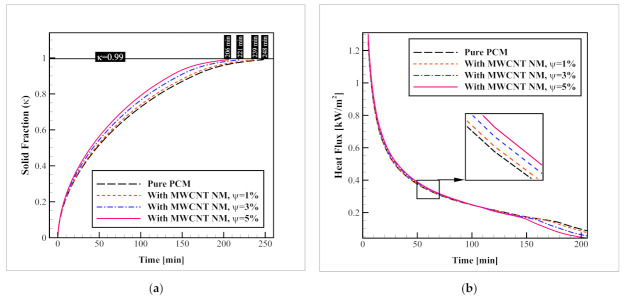
The effects of various volumetric concentrations of the optimal NM type (MWCNT) on (**a**) progression of the average solid fraction; and (**b**) the heat flux passing through the refrigerant tubes.

In the previous section, MWCNT NM was selected as the best carbon-based NM additive for enhancing the phase change process. This section studies the influence of various volume fractions of this NM on the enhancement obtained in presence of these additives. Figure 11 depicts the development of the process with various volume fractions of MWCNT NMs, in terms of solid fraction and heat flux changes. As indicated in the figure, while the complete freezing rate of the pure PCM takes 248 min, the presence of MWCNT NMs with volume fractions of 1, 3, and 5% (ψ = 1, 3, and 5%, respectively) decreases the time to 239, 221, and 206 min, respectively. This means, that the distribution of MWCNT NMs with ψ = 1, 3, and 5% within the PCM improves the solidification speed by 3.62, 10.88, and 16.93%, respectively. A similar trend is observed for the heat flux changes, meaning that as the MWCNT volume fraction increases the heat flux values rise with it. This can be explained by the improved thermal conductivity of the PCM which facilitates the heat transfer from the refrigerant tubes to the PCM and raises the heat flux. It is worth mentioning that for all of these cases, the horizontal and vertical plate-fin networks were used and no geometric alternation was applied.

By comparing these values to that of the benchmark model (690 min), it can be comprehended that the combination of the plate-fin network as well as MWCNT NMs with volume fractions of 1, 3, and 5% can improve the overall solidification process by 65.36, 67.97, and 70.14%, respectively, which are significant improvements.

**Figure 12:**
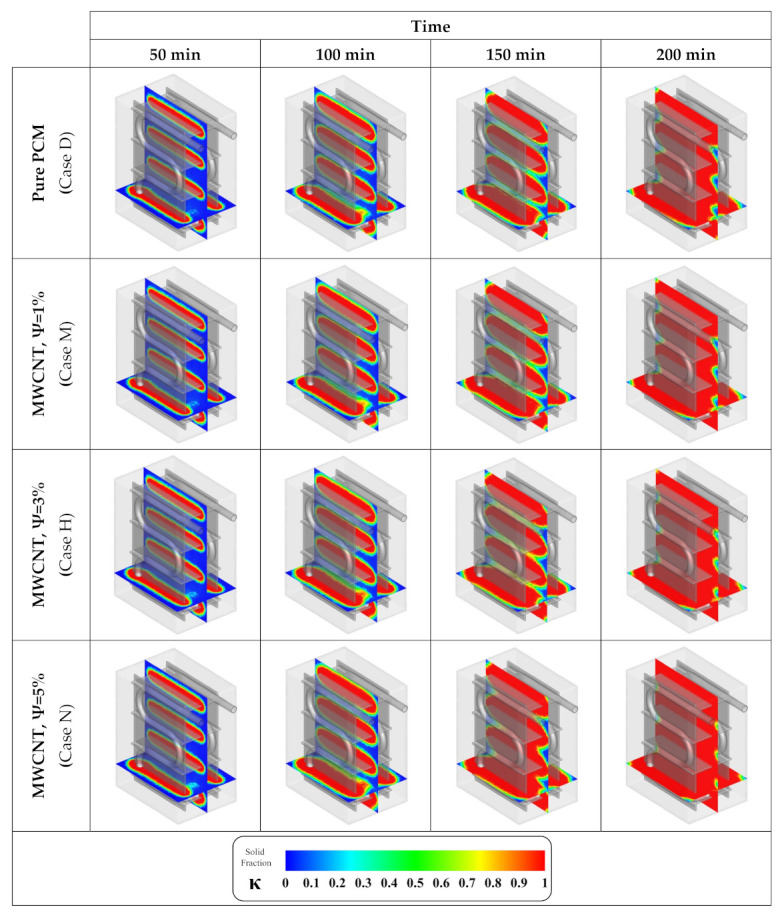
Contours of solid fraction advancement at different volumetric concentrations of MWCNT NMs.

To follow the pattern of changes in the solid fraction contours with various volume fractions of MWCNT NMs, Figure 12 is presented. As shown in the figure, as the MWCNT NM volume fraction rises, the solidification rate is improved which is observed in the form of a more advanced solid fraction in the following solid fraction contours.

## 4. Conclusions and Recommendations

In the present study, a combination of plate-fin networks and carbon-based nanomaterials was introduced as the solution for addressing the sluggish freezing rate of a cuboid-shape cold PCM container. At first, the impacts of three arrangements of plate-fin networks, including, horizontal, vertical, and a combination of these two plate-fin networks were tested and compared with the base model without fins, and then the influence of three different carbon-based NMs, including GO, SWCNT, and MWCNT was evaluated and compared with the cases with CuO NMs, and without NMs, in both identical volume and mass fraction conditions. After selecting the optimal carbon-based NMs, the influence of various NM volume fractions in the range of 0–5% was investigated. The main findings of the research are summarized as follows:

- The horizontal and vertical plate-fin networks present 41.59 and 45.21% of enhancement, while their combination can improve the solidification process by 64.05%. This shows that the horizontal plate-fin arrangement is slightly more influential on the process;
- When carbon-based NMs are compared to the CuO NMs, which are known as the best metal-oxide NMs for this application based on previous studies, in both identical NM volume and mass fraction conditions they present significantly better results in terms of the solidification speed (up to 2.78% and 17.97, at identical NM volume and mass fractions, respectively);
- The comparison of the different carbon-based NMs at identical NM volume fractions is inconclusive, as their results are almost the same in this condition. However, at identical NM mass fractions, MWCNT presents 2.50, and 8.45% better results than GO and SWCNT NMs, respectively. Therefore, among the entire tested NMs, MWCNT NMs present the best results;
- Higher volume fractions of MWCNT NMs, lead to better results when identical HX geometries are used, MWCNTs with NM volume fractions of 1, 3, and 5%, improve the process by 3.62, 10.88, 16.93%, respectively.
- The combination of horizontal and vertical plate-fin networks as well as MWCNT NMs with an NM volume fraction of 5%, declines the solidification time by 70.14%.

For future studies, oblique fins can be tested instead of horizontal and vertical fins. The discharging process of the setup can be evaluated. Moreover, the integration of the current setup with air condition systems in an experimental test can probably yield interesting results and is recommended for future studies.

## Figures and Tables

**Table 1:** The required thermophysical data [32,33].

Property	Copper C11000	GO	CuO	SWCNT	MWCNT	HTF (C_2_H_6_O_2_ 30%vol)	PCM (Pure H_2_O)	
							Liquid	Solid
h_sf_ [J/kg]	-	-	-	-	-	-	334,000	
T_solidus_ [K]	-	-	-	-	-	-	273.15	
T_liquidus_ [K]	-	-	-	-	-	-	273.15	
k [W/m·K]	388	5000	18	6600	3000	242	0.578	1.918
c_p_ [J/kg·K]	385	717	540	425	796	3560	4180	2217
ρ [kg/m^3^]	8933	1800	3970	2600	1600	1054	958.15	
µ [Pa·s]	-	-	-	-	-	0.00620	0.00162	-

**Table 2:** Research variables and groups specified for the analysis.

Research Variable		Case Code	Plate-Fin Arrangement	NM Type	NM Fraction	
					Volume Fraction [%]	Mass Fraction [%]
**Plate-fin arrangement**		A	Without fins	-	0%	
		B	Horizontal fins			
		C	Vertical fins			
		D	Horizontal and vertical fins			
**NM type**	**At identical NM volume fractions**	D	Horizontal and vertical fins	-	3	-
		E		CuO		16.76
		F		GO		5.27
		G		SWCNT		4.09
		H		MWCNT		4.71
	**At identical NM mass fractions**	D		-	-	10
		I		CuO	1.67	
		J		GO	5.81	
		K		SWCNT	7.44	
		L		MWCNT	6.49	
**NM volume fraction**		D		MWCNT	0	0
		M			1	1.59
		H			3	4.71
		N			5	7.76

## Data Availability

The data presented in this study are available on reasonable request from the first corresponding author.
